# Novel Therapeutic Potential of Retinoid-Related Orphan Receptor α in Cardiovascular Diseases

**DOI:** 10.3390/ijms24043462

**Published:** 2023-02-09

**Authors:** Yun Chen, Shu-Ping Zhang, Wei-Wei Gong, Yang-Yang Zheng, Jie-Ru Shen, Xiao Liu, Yun-Hui Gu, Jia-Hai Shi, Guo-Liang Meng

**Affiliations:** 1 Department of Pharmacology, School of Pharmacy, Nantong University, Nantong 226001, China; 2 Nantong Key Laboratory of Translational Medicine in Cardiothoracic Diseases, Research Institution of Translational Medicine in Cardiothoracic Diseases, Nantong University, Nantong 226001, China

**Keywords:** retinoid-related orphan receptor α, staggerer mutant mice, cardiovascular diseases, ligand, agonist

## Abstract

The retinoid-related orphan receptor α (RORα) is one subfamily of nuclear hormone receptors (NRs). This review summarizes the understanding and potential effects of RORα in the cardiovascular system and then analyzes current advances, limitations and challenges, and further strategy for RORα-related drugs in cardiovascular diseases. Besides regulating circadian rhythm, RORα also influences a wide range of physiological and pathological processes in the cardiovascular system, including atherosclerosis, hypoxia or ischemia, myocardial ischemia/reperfusion injury, diabetic cardiomyopathy, hypertension, and myocardial hypertrophy. In terms of mechanism, RORα was involved in the regulation of inflammation, apoptosis, autophagy, oxidative stress, endoplasmic reticulum (ER) stress, and mitochondrial function. Besides natural ligands for RORα, several synthetic RORα agonists or antagonists have been developed. This review mainly summarizes protective roles and possible mechanisms of RORα against cardiovascular diseases. However, there are also several limitations and challenges of current research on RORα, especially the difficulties on the transformability from the bench to the bedside. By the aid of multidisciplinary research, breakthrough progress on RORα-related drugs to combat cardiovascular disorder may appear.

## 1. Introduction

Nuclear hormone receptors (NRs) belong to a family of ligand-regulated transcription factors. The retinoid-related orphan receptors (ROR), including RORα (or NR1F1), RORβ (or NR1F2), and RORγ (or NR1F3) subtypes, are in one NR gene subfamily. RORs bind to DNA in the form of monomers or dimers with distinct tissue expression patterns. In detail, RORα is widely spread in the heart, vessels, bone, lung, skin, kidney, adipose tissue, and cerebellum. RORβ is highly expressed in neurophotoendocrine system, pineal gland, retina, and suprachiasmatic nuclei. RORγ mainly exists in the thymus [1]. Different types of ROR demonstrate distinct effects in various physiological and pathological conditions.

## 2. Physiological Roles of RORα in Cardiovascular System

**Figure 1:**
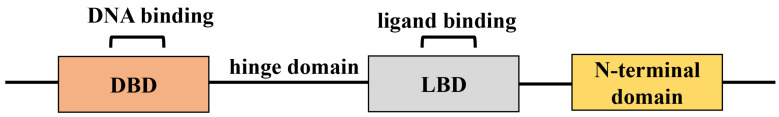
Domain structures of RORα. There are four main domains including a conserved DNA-binding domain (DBD), ligand-binding domain (LBD), hinge domain, and distinct N-terminal domain.

The *RORα* gene is located on human chromosome 15q22.2. There are four main domains including a conserved DNA-binding domain (DBD), ligand-binding domain (LBD), hinge domain, and distinct N-terminal domain (Figure 1) [2]. After the specific ligands bind to LBD, the domain will undergo a conformational change to finally evoke a cascade of downstream events [3] and to play multiple roles in anti-cancer, anti-inflammation, lipid homeostasis maintenance, circadian clock regulation, and so on [2,3]. Therefore, researchers have tried to explore whether RORα can become a potential target, which is beneficial for proposing a novel strategy for disease prevention and treatment.

It is noteworthy that RORα was commonly considered as a critical member to regulate circadian rhythm. Interestingly, RORα was also found in the heart and the vessels [4,5]. Moreover, RORα is known as one kind of the nuclear melatonin receptor. As far as we know, melatonin, as a pineal-gland-secreted hormone, is derived from the amino acid tryptophan. Current evidence has proven that melatonin exerts potent protective effects against several cardiovascular disorders including ischemic heart injury, hypertension, valvular heart diseases, and atherosclerosis [6,7,8]. All these studies suggest that RORα might offer potential therapeutic approaches for cardiovascular diseases.

This review summarizes the understanding and potential effects of RORα in the cardiovascular system and then analyzes current advances, limitations and challenges, and further strategy for RORα-related drugs in cardiovascular diseases.

## 3. RORα-Deficiency Mice—Staggerer Mutant Mice

It is interesting to find that if there is a deletion or mutation in the *RORα* gene, translation of the LBD will be prevented, and the activity of RORα will be deficient. These mice harboring a germline mutation encoding a truncated and globally nonfunctional RORα will manifest as ataxia associated with cerebellar degeneration due to a cell-autonomous defect in the development of Purkinje cells. The Purkinje cells demonstrate immature morphology, disordered synaptic arrangement, abnormal biochemical characteristics, irregular gene expression, and decreased cell numbers. In addition, dendritic atrophy and cell loss were accelerated in RORα-deficient homozygous mice [9]. The mice demonstrating the above obvious phenotype were named spontaneous staggerer (sg/sg) mutant mice, which became a common model to elucidate the role of the RORα signaling pathway. Gradually, a variety of other phenotypes was also found in sg/sg mutant mice, including smooth muscle cell dysfunction and enhanced susceptibility to atherosclerosis and myocardial hypertrophy [10,11]. The sg/sg mutant mice facilitate the study of the role of RORα in the cardiovascular system.

However, the sg/sg mutant mice have several disadvantages. First, common granular feed is too hard for sg/sg mutant mice to digest and absorb, so all feed has to be made into a mushy diet throughout the whole life. In addition, due to staggering during walking, it is preferred that they are kept in a cage alone to avoid trampling and extrusion. Third, the period to obtain homozygotes is relatively long because of the absent reproductive ability in sg/sg mutant mice, which delays the experimental progress. Furthermore, because of several defects of basic functions, mortality is prone to be higher than wild mice, especially during disease model making. Therefore, some novel RORα-deficient mice with better life quality and greater reproducibility are urgently needed.

## 4. Protective Roles of RORα against Cardiovascular Diseases

### 4.1. Atherosclerosis

It evoked scientists’ interest that RORα existed in atherosclerotic plaques as well as aortic smooth muscle cells and endothelial cells. RORα isoforms may vary in different cells or tissues. In detail, aortic smooth muscle cells mainly expressed RORα1, and endothelial cells dominantly expressed RORα4, while RORα2 and RORα3 have not been detected in the above two cells [12]. However, the roles and exact mechanisms of different RORα isoforms remains unclear, which bring difficulties in the design and optimization of drugs specific to RORα subtype.

Staggerer mutant mice have demonstrated higher susceptibility to suffer from atherosclerosis with dyslipidemia and exhibited more severe atherosclerotic plaques [13]. Interestingly, cholesterol and related derivatives have been identified as natural ligands of RORα [14]. RORα appears to participate in the regulation of plasma cholesterol levels as well as apolipoprotein (apo)A-I and apoC-III gene expression [15]. In human monocytic cell line (THP-1) and human umbilical vein endothelial cell line (HUVEC), RORα bound to RORα response elements in the promoter of cytochrome P450 family 19 subfamily A member 1 (CYP19A1), migration inhibitory factor (MIF), and ATP-binding cassette transporter A1 (ABCA1), respectively [16]. Indeed, RORα ligands CPG 52608 or SR1001 differentially regulated the mRNA expression of CYP19A1, MIF, and ABCA1. In consideration that cholesterol biosynthesis can be blocked by statins to attenuate atherosclerosis, simvastatin was applied to verify that lowering cholesterol inhibited the expression of the above three target genes. Moreover, the preventive effect of simvastatin against target genes was partially restored by CPG 52608 or SR1001 [16]. These data suggest that CYP19A1, MIF, and ABCA1 are direct target genes of RORα, which provides evidence that RORα regulates cholesterol synthesis, inflammation, and cholesterol efflux in atherosclerosis.

In skeletal muscle cells, RORα bound to the promoter of carnitine palmitoyltransferase-1 (CPT-1) as well as caveolin-3 and was co-activated by p300 and peroxisome proliferators activated receptor-γ (PPARγ) co-activator-1 (PGC-1). Furthermore, over-expression of exogenous dominant negative RORα in skeletal muscle cells repressed endogenous levels of RORα mRNAs and reduced transcription of CPT-1 and caveolin-3. Conversely, RORα agonists enhanced both CPT-1 and caveolin-3 expression to promote fatty acid catabolism in skeletal muscle [17]. In short, RORα activators may have therapeutic prospects in the treatment of atherosclerosis. In human monocyte-derived macrophages with interferon-γ (IFN-γ) or lipopolysaccharide (LPS) stimulation, RORα agonists regulated macrophage polarization via AMP-activated protein kinase α (AMPKα) AMPK-signal transducers and activators of the transcription (STAT) pathway in a RORα-dependent manner [18]. Taken together, there is no definitive conclusion about the exact target genes of RORα during atherosclerosis, which might depend on cell types, stimulus properties, stimulation time, surrounding microenvironment, and so on.

RORα agonist has shown a powerful anti-atherosclerosis effect. RORα agonist prevented vulnerable plaque rupture and suppressed intraplaque hemorrhage in renovascular hypertension combined with low shear stress of hypercholesterolemic ApoE^−/−^ mice [18]. High dosages of RORα agonist also enhanced atherosclerotic plaque stability via prolyl-4-hydroxylase α1 (P4Hα1) up-regulation in ApoE^−/−^ mice when placing a perivascular collar on the right common carotid artery [19]. Infusion of adenovirus encoding RORα into arteries inhibited neointima formation in rats with balloon injury through AMPK-induced mammalian target of rapamycin (mTOR) suppression. In terms of mechanism, RORα activation inhibited vascular smooth muscle cells proliferation via modulating the expression of cell-cycle-regulating factors including p53, p27, and cyclin D [20]. Enhanced RORα was able to be bound to angiopoietin-like 4 (ANGPTL4) promoter in mesenchymal stem cells (MSCs) to exert anti-inflammatory effects against macrophages [21]. In addition, RORα activation attenuated mitophagy activation and NLR family pyrin domain-containing protein 3 (NLRP3) inflammasome [22], alleviated endothelial cell pyroptosis [23], and ameliorated vascular endothelial dysfunction [24]. These data demonstrate that RORα was involved in atherosclerosis-related gene expression regulation, indicating that RORα is a negative factor of atherosclerosis, which offers a potential therapeutic approach for the treatment of atherosclerosis.

### 4.2. Hypoxia or Ischemia

After ligation of the right femoral artery, there were higher angiographic scores for capillary density, richer perfusion, and more extensive angiogenesis within the ischemic hindlimb in the staggerer mouse than that in the wild-type (WT) mice. The increased angiogenesis after ischemia might be attributed to RORα-absence-associated exacerbation of inflammatory cytokines [25]. This seems to suggest that RORα might be a potent negative regulator of ischemia-induced angiogenesis.

However, later research verified a contradictory role of RORα in other vessels subjected to hypoxia or ischemia. It is commonly known that hypoxia-inducible factor 1α (HIF-1α) is primarily involved in the adapting of oxygen-level variation with hypoxia or ischemia stimulation. One study confirmed that both the transcriptional activity and protein level of HIF-1α were enhanced after RORα exogenous introduction. Two different ligands of RORα stimulation similarly enhanced HIF-1α expression and promoted transcriptional activity, which was blunted by RORα knock-down with RNA interference. Furthermore, either adenovirus encoding RORα infection or RORα ligands administration increased the formation of capillary tubes in human umbilical vascular endothelial cells [26]. The different effects of RORα on angiogenesis may be attributed to distinct ischemic time or degree, vascular characteristics, pathophysiological state, ligands level, and so on. Therefore, the real influence of RORα on angiogenesis during hypoxia or ischemia might not be simple promotion or inhibition, which should be further investigated.

In addition, circadian rhythm disruption or decrease in levels of circadian hormones increases ischemic heart disease risk [27]. Indeed, RORs was known to be pivotally involved in circadian rhythm regulation. Therefore, the functional roles of RORs in the heart have been gradually clarified. For example, melatonin significantly improved cardiac dysfunction and alleviated myocardial remodeling in left anterior descending (LAD) coronary artery ligation-induced myocardial infarction (MI). Mammalian Ste20-like kinase 1 (Mst1) is a core molecular in the mammalian hippo pathway, which enhances apoptosis and suppresses autophagy to mediate heart failure after infarction. Melatonin blocked Mst1 phosphorylation but elevated sirtuin 1 (SIRT1) expression after MI. Moreover, above protective effects on MI were abolished by Mst1 deficiency. This suggests that melatonin promoted autophagy, inhibited apoptosis, and maintained mitochondrial integrity and biogenesis at least in part via Mst1/SIRT1 signaling [28]. Another example is that melatonin supplement showed a synergetic effect to improve heart function and enhance functional survival of adipose-derived mesenchymal stem cells (AD-MSCs) in the heart after LAD coronary artery ligation. In terms of mechanism, melatonin decreased the acetylation level of forkhead box O1 (FoxO1), p53, and nuclear factor kappa-B (NF-κB) via SIRT1 enhancement to ameliorate inflammation, apoptosis, and oxidative stress [29]. These studies provide a novel insight for RORα in amplification of hypoxia signaling and propose a potential strategy of RORα ligands for the therapy of hypoxia-related cardiovascular diseases.

### 4.3. Myocardial Ischemia/Reperfusion Injury

Actually, the ischemic myocardium will suffer more serious myocardial ischemia-reperfusion injury (MI/R) if the blood supply is restored after ischemia for a short time. Evidence showed that RORα expression was downregulated after MI/R. Furthermore, compared with WT mice, myocardial infarct area, cardiomyocyte apoptosis, and systolic dysfunction were significantly promoted in staggerer mutant mice. In terms of mechanism, RORα deficiency promoted endoplasmic reticulum (ER) stress, aggravated mitochondrial damage and autophagy dysfunction, and enhanced myocardial oxidative or nitrative stress. By contrary, cardiomyocyte-specific RORα over-expression mice became less vulnerable to MI/R injury [27]. The study suggests that RORα is possibly a novel endogenous defender against MI/R injury.

Given the emerging evidence of melatonin as a potential RORα activator, 15 eligible studies with 211 animals confirmed that RORα activation exerted cardioprotection against MI/R injury in preclinical studies [30]. In rats, RORα agonist inhibited oxidative stress, improved cardiac function, suppressed infarct size, reduced apoptosis index, and inhibited the release of serum creatine kinase and lactate dehydrogenase in MI/R. The above protective effects were blocked by SIRT1 inhibitor EX527 or melatonin receptor antagonist luzindole [4,31]. A similar protective effect was also found in mice with LAD ligation for 50 min followed by reperfusion for 4 h in vivo and global ischemia for 40 min followed by reperfusion for 45 min in vitro, all of which were not changed if glutathione peroxidase 1(Gpx1) was deficient [32]. This indicates that SIRT1 and melatonin receptor but not Gpx1 may be particularly involved in the protective effects of RORα activation against MI/R injury. In addition, RORα agonist augmented the protective effect against myocardial infarct by remote ischemic perconditioning (RIPerC) in both non-pinealectomized and pinealectomized mice. The study also indicated that physiological release or pharmacological RORα activation ameliorated myocardial ischemia-reperfusion injury by modulating cytochrome b-245 beta chain (Cybb) gene, Fas gene, and NF-κB [33]. In summary, RORα is a potential endogenous cardioprotective receptor against ischemic heart injury.

The damage of MI/R was significantly aggravated, and the survival rate was reduced if the circulating concentration of endogenous melatonin was decreased by pinealectomy [34]. Due to the natural decrease of endogenous melatonin level in elderly people, the aged may be vulnerable to more serious heart damage. Considering the high efficacy to MI/R and low toxicity, appropriate supplement of melatonin seems to have advantages over other antioxidants in terms of ameliorating MI/R. However, the effects of melatonin on patients with acute myocardial infarction were not identical in different studies. A single-center trial with small samples showed that there was nocturnal melatonin disorder in patients with myocardial infarction [35]. Surprisingly, a multicenter, randomized, double-blind, placebo-controlled study found that there was no significance on infarct size during MI/R injury after melatonin treatment in patients with primary percutaneous coronary intervention. Even worse, melatonin may aggravate cardiac remodeling and delay the recovery of cardiac function [36]. Then, the patients were divided into different subgroups according to the pain-to-balloon time in further statistical analysis. This demonstrated that melatonin was only effective to reduce myocardial infarct size if the time from symptom onset to balloon was less than 136 min. Otherwise, melatonin had an opposite effect to increase myocardial infarct size in the long pain-to-balloon-time group [37]. In summary, the above contradictory benefits of melatonin on MI/R injury were possibly attributed to different pain-to-balloon times. However, the present sample size in each subgroup was small, and further studies are needed to assess whether melatonin improves the clinical prognosis of MI/R.

### 4.4. Diabetic Cardiomyopathy

Diabetic cardiomyopathy (DCM) is a common complication with cardiac structural and functional disorder to increase morbidity and mortality in diabetes, which is independent of valvular heart disease, hypertension, coronary atherosclerosis, and other diseases. Previous studies have confirmed that RORα level was downregulated in the myocardium of diabetic mice. Moreover, RORα-deficient mice exhibited more serious myocardial hypertrophy and worse cardiac function during the development of diabetic cardiomyopathy, which may be attributed to apoptosis augment, autophagy dysfunction, and oxidative stress enhancement. Furthermore, cardiomyocyte-specific RORα transgenic (TG) mice showed attenuated cardiac function impairment and myocardial damage in diabetic mice [38]. These studies demonstrate the potential protective role of RORα against DCM.

Accordingly, melatonin, a natural RORα agonist, in the nocturnal level of both the circulatory system and myocardium was significantly decreased in streptozotocin (STZ)-induced and high-fat-diet-fed diabetic rats. Long-term RORα agonist supplementary suppressed mitochondrial fission and promoted mitochondrial biogenesis and mitophagy via sirtuin 6 (SIRT6)/AMPK/PGC-1α/protein kinase B (AKT) axis to delay the progression of DCM [39]. This study implicated that protective effects in targeting mitochondrial quality by RORα activation are a promising strategy for DCM treatment.

**Figure 2:**
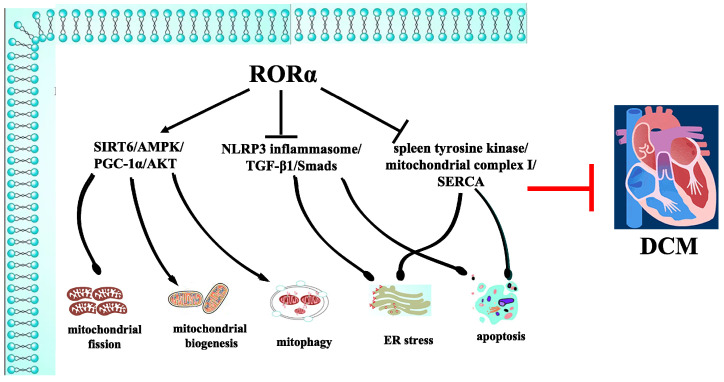
Molecular mechanisms and cellular pathways involved in RORα’s protective roles against DCM. RORα agonist suppressed mitochondrial fission and promoted mitochondrial biogenesis and mitophagy via SIRT6/AMPK/PGC-1α/AKT axis to attenuate DCM. RORα activation also ameliorated cardiac ER stress and apoptosis via inhibiting NLRP3 inflammasome activation and blocking TGF-β_1_/Smads signaling in DCM. RORα activation also blocked spleen tyrosine kinase/mitochondrial complex I/SERCA axis to alleviate DCM.

Besides regulating mitochondrial biogenesis, RORα was also involved in rescuing the impaired mitophagy via inhibiting Mst1 phosphorylation-mediated parkin translocation in the heart of DCM [40]. In addition, RORα agonist corrected blood glucose and lipid metabolism disorder, suppressed extrinsic and intrinsic apoptotic pathways, modulated mitochondrial integrity and biogenesis, and attenuated diabetic myocardium injury in STZ-administrated rats [41,42,43,44,45]. RORα activation may also ameliorate cardiac ER stress-induced apoptosis and alleviate cardiac fibrosis via inhibiting NLRP3 inflammasome activation and blocking transforming growth factor (TGF)-β_1_/Smads signaling in DCM [44,46]. It is worth mentioning that another group proposed spleen tyrosine kinase/mitochondrial complex I/sarcoendoplasmic reticulum (SR) calcium transport ATPase (SERCA) axis activation as a novel pathway contributing to DCM, which was able to be blocked by RORα activation via alleviating caspase-9-involved mitochondrial apoptosis and caspase-12-related ER apoptosis-mediated cardiomyocyte damage [47]. Another example was synthetic RORα agonist SR1078, showing a powerful ability to alleviate STZ-induced DCM in mice [38]. On the other hand, RORα inhibitor SR3335 further exacerbated cardiac damage in diabetic mice [38]. Still, detailed molecular mechanisms and cellular pathways involved in RORα’s protective roles against DCM remain unclear (Figure 2). All of the above research demonstrates that both pharmacological activation of RORα by specific ligands and genetical manipulation of restoring RORα by cardiac over-expression may exert a beneficial effect against DCM. Thus, RORα-related agents may be a promising and potential candidate to ameliorate DCM.

### 4.5. Hypertension

RORα mRNA expression was decreased in Th17 cells from angiotensin II (Ang II)-induced mice with hypertension. This suggests that there may be a correlation between RORα and hypertension [48]. Another research showed that maternal RORα activation prevented postnatal dexamethasone-induced programmed hypertension [49]. This suggests a distinctive therapeutic strategy for long-term protection against glucocorticoids-induced programmed hypertension in premature babies despite the unknown mechanism.

However, RORα activation exerted no significant effects on blood pressure in RORα and ApoE-double-knocked-out mice with renovascular hypertension by ligating the left renal artery [18]. Furthermore, circadian genes were considered to have a vital role in maintaining the circadian rhythm of the cardiovascular system, including diurnal variation of blood pressure. A genetic association study of young-onset hypertension demonstrated that rs10519096 in RORα was significantly associated with the non-dipper phenotype in 372 young hypertensive patients [50]. Therefore, the causal relationship between RORα and hypertension is not known well. The potential clinical application prospect of RORα for other common types of hypertension needs to be further verified and clarified.

### 4.6. Myocardial Hypertrophy

Pathological myocardial hypertrophy is initially a compensatory response to substantial pressure or volume overload and neurohormonal activation, which is prone to heart failure if lasting for a long time. RORα was significantly decreased in human and mouse models with pathologically hypertrophy but not in mice with swimming-induced physiologically hypertrophy. RORα agonist administration attenuated transverse aortic constriction (TAC)-induced pathological hypertrophy and suppressed myocardial oxidative stress. However, the above protective effects against pathological myocardial hypertrophy were unavailable in RORα-deficient mice. Further study found that RORα agonist combined with RORα response elements in the promoter of manganese-dependent superoxide dismutase (MnSOD) induces MnSOD transactivation and thus combats myocardial hypertrophy. Furthermore, MnSOD over-expression attenuated myocardial hypertrophy in RORα-deficient mice, while knocking down MnSOD blunted the preventive effect against myocardial hypertrophy [5]. Another group found that RORα abundance was seriously impaired in mouse and human models with heart failure. Furthermore, there was more aggravated myocardial hypertrophy in RORα-deficient mice than in WT mice with Ang II continuous perfusion for 2 weeks. Similar results were obtained in cultured neonatal rat ventricular myocytes of gain- and loss-of-function experiments. Further research confirmed that RORα function deficiency elevated interleukin 6 (IL-6) expression, promoted STAT3 activation, deteriorated mitochondrial function, aggravated oxidative stress, decreased total cardiomyocyte number, and finally exacerbated Ang II-induced myocardial hypertrophy [11,51]. Similar exacerbation on myocardial hypertrophy was also exhibited in RORα-deficient mice with high-fat diet (HFD), which was possibly attributed to AMPK-PGC1α signaling pathway inhibition, while the above damage was restored by RORα over-expression [52]. In addition, RORα agonist effectively attenuated fine particulate matter 2.5 (PM_2.5_)-induced cardiac dysfunction and remodeling via surtuin 3 (SIRT3)-mediated MnSOD deacetylation [53]. It is worth noting that hypertrophy can be adaptive and pathological, and hypertrophy itself does not always lead to failure, especially during early phases of hypertrophy. Heart failure is the result of the destruction of myocytes leading to decreased contraction or contractility. Therefore, the respective roles of RORα in the development of the above two pathologies and mechanisms need to be known for developing potential therapy targets for prevention and treatment of pathological hypertrophy of the myocardium.

However, RORα agonist is not appealing in clinic because of the short-lasting time after oral administration. A novel biocompatible dual-targeting nanoagent was developed with cardiac homing peptide (CHP) and superparamagnetic iron oxide nanoparticles (SPIONs). Only a low dosage of melatonin carried by the biocompatible dual-targeting nanoagent (CHP-mel@SPIONs) was able to be accumulated in the heart to ameliorate TAC-induced myocardial hypertrophy [54]. Obviously, this novel compound carries less but transports more drugs to the target organs, which provides an exemplary example for the research and development of RORα-related drugs in the future. Some novel drug delivery and transportation strategies are urgently needed to develop ideal candidates based on RORα.

## 5. RORα-Related Compounds

Sterols are commonly considered as natural ligands for RORα [55]. Crystal structure analysis has shown that cholesterol and related intermediates reversibly bind to the LBD of RORα [56,57]. Besides cholesterol, cholesterol-3-*O*-sulphate and 24 *S*-hydroxycholestrol also regulated transcriptional activity and target gene expressions of RORα [3,14]. Interestingly, a natural plant sterol called neoruscogenin was screened from a library of 12,000 plant extract fractions as an RORα agonist [58]. Additionally, Nobiletin is a natural compound that directly binds to and activates RORα, modulating circadian rhythms and showing robust in vivo efficacies to combat clock-associated pathophysiologies and age-related decline [59]. One latest study confirmed that diosgenin acted as a direct ligand and inverse agonist of RORα, which is a key transcription factor involved in Th17 cell differentiation and metabolism [60]. Another group underscored the screening of a large combinatorial library of 1,5-disubstituted acylated 2-amino-4,5-dihydroimidazoles using a demonstrated synthetic and screening approach and the utility of the positional scanning libraries strategy for the rapid identification of a novel class of RORα inhibitors. They identified a novel compound with 5.3 µM IC50 against RORα [61]. All these data suggest that RORα is a potential target for various diseases.

The above series of studies confirms the protective effects of melatonin against cardiovascular diseases. Undoubtedly, RORα is inextricably linked with melatonin in expressions, functions, and possible mechanisms. In addition, studies have shown that similar to RORα ligands, melatonin regulates the transcriptional activity of RORα. Nevertheless, some studies supporting the above interactions have been withdrawn recently. Surprisingly, crystallographic evidence did not support RORα as a nuclear receptor for melatonin. Some other studies have also demonstrated that melatonin indirectly regulates RORα activity without direct binding to RORα [62,63,64]. Regardless, although whether RORα as one nuclear receptor of melatonin is still controversial, melatonin did activate RORα, and numerous studies still attribute melatonin’s effects to RORα. Indeed, melatonin may act through RORα, but it has its own receptors such as melatonin receptor G protein-coupled receptors MT_1_ and MT_2_, which are involved in many protective actions on the cardiovascular system. Therefore, there is still debate as to whether melatonin is a real RORα ligand or agonist. The questions that need to be answered are which protective actions are mediated via RORα and which are not. therefore, the relationship between melatonin and RORα needs to be clarified better in the future.

In addition to natural ligands, thiazolindinedione (CGP 52608) was the first described synthetic ligand for RORα. However, the ability to activate RORα was weak [65]. T0901317, an inverse agonist, was the first validated synthetic ligand to bind to both RORα and RORγ receptors and then regulate their functions [66]. Because of the nonspecific binding of T0901317 to several nuclear receptors, T0901317 was regarded as a starting point for RORα-selective compound development. Similar to T0901317, SR1078 is another non-specific agonist of RORα and RORγ [67]. Based on T0901317 and SR1078 scaffolds, SR3335 was discovered after additional modifications, which was the first potent RORα-specific inverse agonist with acceptable pharmacokinetic properties [68]. However, all the above synthetic ROR ligands were limited to basic proof-of-concept in vivo until the discovery of SR1001. SR1001 firstly demonstrated the real role of synthetic RORα ligand in the mouse. This compound directly binds to RORα and RORγ and acts as an inverse agonist to suppress RORα and RORγ reporter activity [69]. The above characterization of endogenous or synthetic ligands has opened up the possibility of targeting RORα to treat several diseases. Nevertheless, RORα-related ligands are mostly being studied in fundamental research, and there is still a long way to go for practical application.

## 6. Limitations and Challenges of Current Research on RORα

Current research on RORα also has several limitations, which brings obstacles to further development of related drugs targeting RORα. First, the roles of RORα in the embryonic development of cardiovascular system as well as functions of various parts of this system have not been clarified. There are few reports on roles of RORα in cardiac electrophysiology including action potential, calcium dynamics, etc. Second, many experiments at the cellular level are often too simplistic to be translated into clinical practice. Then, in animal level studies, the most common RORα-deficiency mice, namely staggerer mutant mice, are subjected to the global disruption of RORα, which ignores the specific contribution of RORα in different cell types. Additionally, there are only sporadic reports on cardiac-specific RORα over-expression. This suggests that cell-specific and inducible RORα-knockout mice, especially cardiomyocyte or endothelial-cell-specific RORα-knockout mice, are urgently needed, which will help us delineate the role of RORα in the cardiovascular system.

In addition, there may be great differences between basic research and clinic investigations. Animal experiments invariably deal with normal or otherwise healthy rodents initially. The evaluation criteria for the efficacy of pharmaceutical treatment after RORα-related drugs treatment varies greatly, which may achieve inconsistent or even paradox conclusions. An example is that infarct size was commonly used as the primary end-point during short-term MI/R in mice, while this is impossible to be assessed in humans. Notably, the mortality and the morbidity, more vital than infarct areas, were chosen as the clinical end-points. This inconsistency contributed to the significant improvement on MI/R by RORα activation in mice but not in humans [30,36]. Although a number of valuable lessons can be taken from these cardioprotection trials to optimize the drug therapy, the above definite differences inevitably bring difficulties to the translation from the bench to the bedside.

Interestingly, all four isoforms including RORα1–4 are expressed in humans, whereas only RORα1 and RORα4 are expressed in mice [2]. The differences in the expression and function of these isomers between animals and humans hinder the epitaxial significance of the potential clinical transformation. Furthermore, the effects of RORα excitation, sometimes even exhibiting biphasic effects on angiogenesis and ischemia-reperfusion injury, remain unclear [25,26,70]. This might be related to different administration manners, a strict time window, and effective blood drug concentration, which brings challenges to the follow-up research and development of RORα-related drugs.

## 7. Further Perspectives and Strategies for RORα-Related Drugs

**Figure 3:**
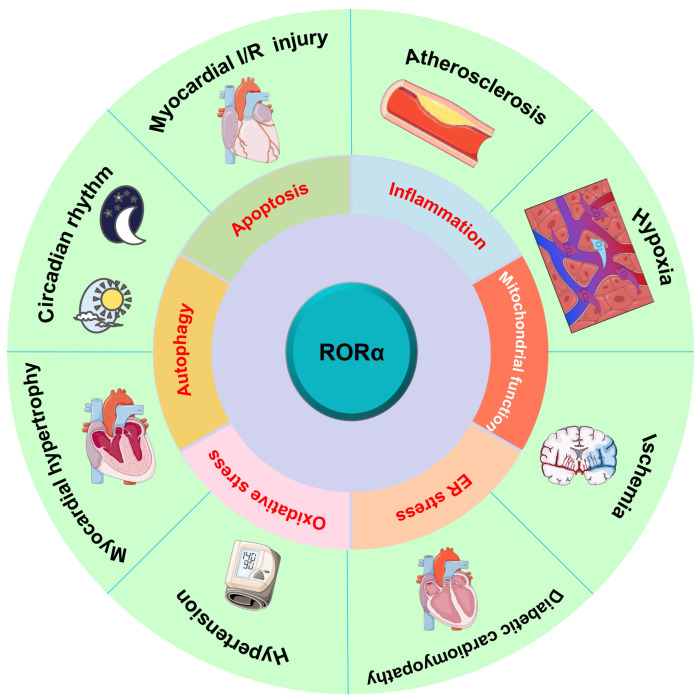
Potential protective effects and mechanism of RORα on cardiovascular diseases. Besides circadian rhythm regulation, RORα influences a wide range of physiological and pathological processes in the cardiovascular system, including atherosclerosis, hypoxia, myocardial ischemia/reperfusion (I/R) injury, diabetic cardiomyopathy (DCM), hypertension, and myocardial hypertrophy. In terms of mechanism, RORα is involved in the regulation of inflammation, apoptosis, autophagy, oxidative stress, endoplasmic reticulum (ER) stress, and mitochondrial function.

In recent years, RORα was considered as an important therapeutic target in more and more diseases and research fields. With the deeper understanding of pathophysiological function and mechanism of RORα in the cardiovascular system and not just in circadian rhythm regulation, several independent studies have confirmed that RORα has a potential protective effect against a variety of cardiovascular diseases as a negative factor, which offers novel therapeutic approaches for cardiovascular diseases including atherosclerosis, hypoxia, myocardial ischemia/reperfusion injury, diabetic cardiomyopathy, hypertension, and myocardial hypertrophy. In terms of mechanism, RORα is involved in the regulation of inflammation, apoptosis, autophagy, oxidative stress, ER stress, and mitochondrial function (Figure 3). Furthermore, there are endogenous ligands for RORα in vivo, suggesting that exogenous agonists or antagonists without serious adverse reactions are possibly available to regulate the expression or activity of RORα by pharmacological means and, finally, to make the RORα level meet the physiological needs of the body. It is beneficial to maintain the steady state of the cardiovascular system. In view of its high efficiency and low toxicity, there are great opportunities for the research of RORα-related drugs.

Due to different role of RORα in various diseases, RORα activation or inhibition is preferred depending on specific pathophysiological state. Therefore, on the basis of known ligands, agonists, or antagonists, an “RORα regulator” with better druggability and selectivity will be created after the structure is modified or optimized via pharmacochemical strategies such as virtual screening and computer-aided drug design. These candidates of novel RORα-related active compounds are of great significance in the treatment of cardiovascular diseases.

On the other hand, likely benefitting from the rapid development of materials science and pharmaceutical pharmaceutics, the solubility, stability, bioavailability, and absorption-distribution-metabolism-excretion (ADME) of RORα-related drugs are expected to be improved. In parallel, therapeutic strategies to regulate RORα activity would be augmented by site-specific delivery, especially for the recently developed dual-targeting nanoagent enabling delivery of RORα modulators to the heart and disease-associated blood vessels, thereby maximizing therapeutic effects but minimizing the side effects. Moreover, modern nanomedicine-integrated artificial intelligence offers opportunities for personalized treatment. Using the reticuloendothelial system will be beneficial to avoid macrophagy system capture, prolong blood circulation time, and achieve long-term effect. These potential strategies offer a good opportunity to provide targeted treatment for cardiovascular diseases by relevant RORα drugs.

**Figure 4:**
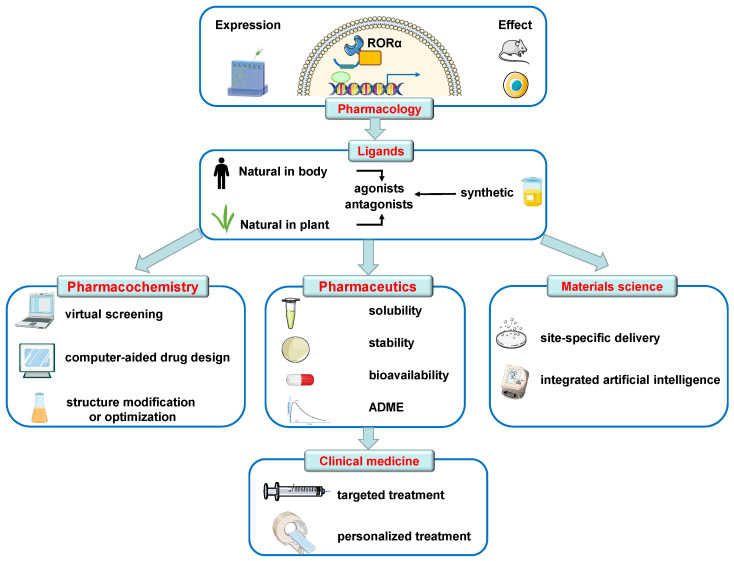
Further perspectives and strategies for RORα-related drugs. With the deeper understanding of the pathophysiological function and mechanisms of RORα in the cardiovascular system, protective pharmacological effects against a variety of cardiovascular diseases as a negative factor have been confirmed. Furthermore, exogenous agonists or antagonists without serious adverse reactions are possibly available to regulate the expression or activity of RORα by pharmacological means. On the basis of known ligands, agonists, or antagonists, an “RORα regulator” with better druggability and selectivity will be created via pharmacochemical strategies such as virtual screening and computer-aided drug design. On the other hand, likely benefitting from the rapid development of materials science and pharmaceutical pharmaceutics, the solubility, stability, bioavailability, and absorption-distribution-metabolism-excretion (ADME) of RORα-related drugs are expected to be improved. In parallel, therapeutic strategies to regulate RORα activity would be augmented by site-specific delivery, especially for the recently developed dual-targeting nanoagent enabling delivery of RORα modulators to the heart and disease-associated blood vessels. Moreover, modern nanomedicine-integrated artificial intelligence offers opportunities for personalized treatment. Using the reticuloendothelial system will be beneficial to avoid macrophagy system capture, prolong blood circulation time, and achieve long-term effect. These potential strategies offer a good opportunity to provide targeted treatment for cardiovascular diseases by relevant RORα drugs in a clinic setting.

Overall, by the aid of multidisciplinary research involving materials science, clinical medicine, pharmacology, pharmaceutical chemistry, and pharmaceutical pharmaceutics, breakthrough progress on RORα-related drugs to combat cardiovascular disorder may appear (Figure 4). Further characterization of the mechanisms of RORα action will not only identify RORα target genes and provide additional insight into their normal physiological functions but also determine their protective roles in cardiovascular diseases.

## Data Availability

Not applicable.
